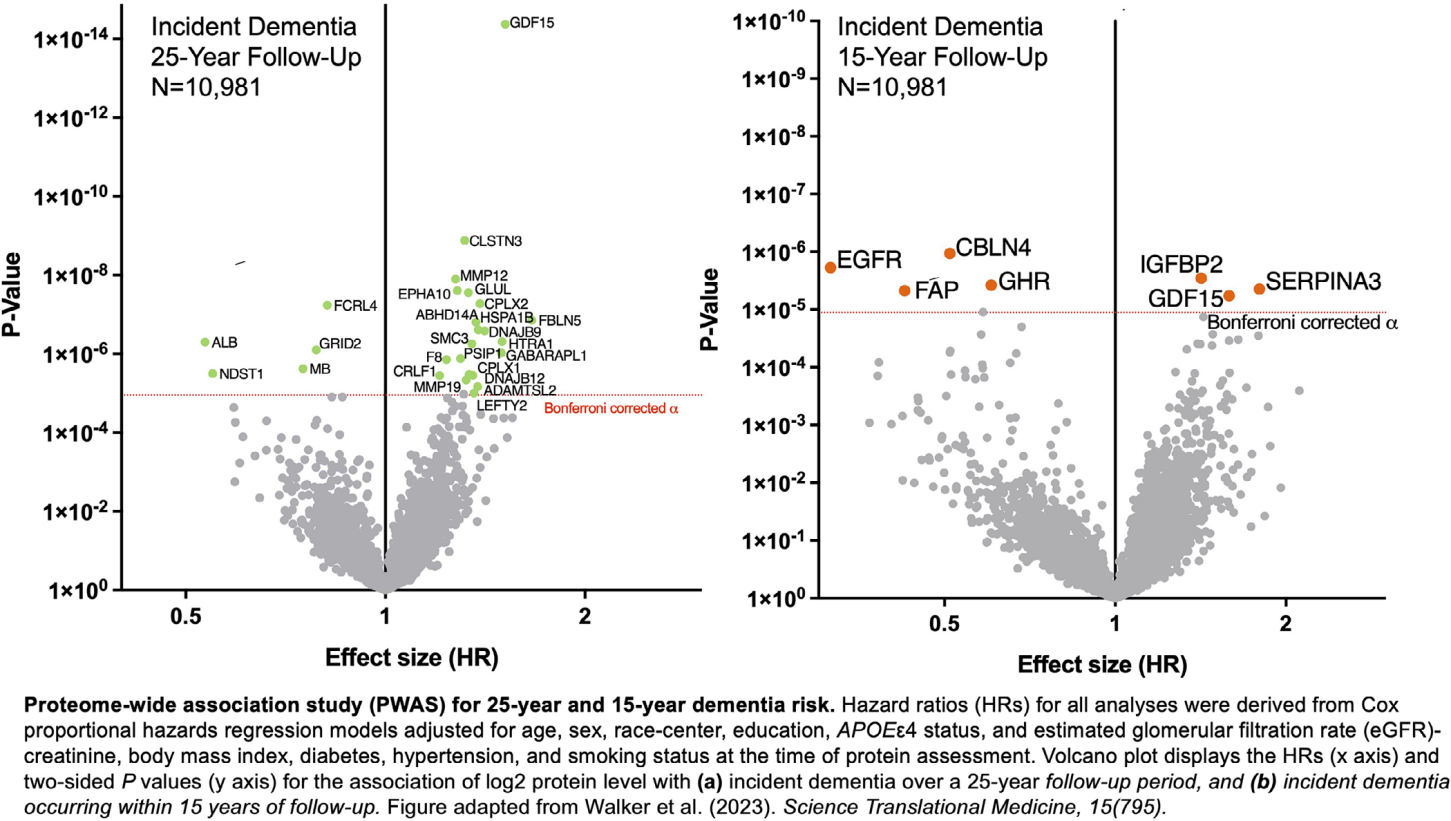# Plasma proteome‐wide analysis of dementia risk mechanistically implicates synaptic biomarkers

**DOI:** 10.1002/alz70856_105743

**Published:** 2026-01-10

**Authors:** Keenan A. Walker, Jingsha Chen, Liu Shi, Yunju Yang, Myriam Fornage, Linda Zhou, Pascal Schlosser, Aditya Surapaneni, Morgan E Grams, Michael R. Duggan, Zhongsheng Peng, Gabriela T. Gomez, Adrienne Tin, Ron C. Hoogeveen, Kevin J. Sullivan, Peter Ganz, Joni V Lindbohm, Mika Kivimaki, Alejo J Nevado‐Holgado, Noel Buckley, Rebecca F. Gottesman, Thomas H. Mosley, Eric Boerwinkle, Christie M Ballantyne, Josef Coresh

**Affiliations:** ^1^ National Institute of Aging Intramural Research Program, National Institutes of Health, Bethesda, MD, USA; ^2^ Department of Epidemiology, Johns Hopkins University Bloomberg School of Public Health, Baltimore, MD, USA; ^3^ Heptares Therapeutics Ltd, Cambridge, United Kingdom; ^4^ The University of Texas Health Science Center at Houston, Houston, TX, USA; ^5^ Brown Foundation Institute of Molecular Medicine, McGovern Medical School; School of Public Health, The University of Texas Health Science Center, Houston, TX, USA; ^6^ Department of Epidemiology, Johns Hopkins Bloomberg School of Public Health, Baltimore, MD, USA; ^7^ Johns Hopkins Bloomberg School of Public Health, Baltimore, MD, USA; ^8^ New York University, New York, NY, USA; ^9^ Laboratory of Behavioral Neuroscience, National Institute on Aging, Intramural Research Program, Baltimore, MD, USA; ^10^ Department of Neurology, Johns Hopkins University School of Medicine, Baltimore, MD, USA; ^11^ University of Mississippi Medical Center, Jackson, MS, USA; ^12^ Baylor College of Medicine, Houston, TX, USA; ^13^ University of Mississippi Medical Center, The MIND Center, Jackson, MS, USA; ^14^ UCSF Medical Center, San Francisco, CA, USA; ^15^ Department of Epidemiology and Public Health, University College London, London, United Kingdom; ^16^ Epidemiology and Health Care, University College London, London, United Kingdom; ^17^ Centre for Artificial Intelligence in Precision Medicines, Oxford, United Kingdom; ^18^ Department of Psychiatry, University of Oxford, Oxford, United Kingdom; ^19^ National Institute of Neurological Disorders & Stroke, Bethesda, MD, USA; ^20^ Departments of Population Health and Medicine, New York University Grossman School of Medicine, New York, NY, USA

## Abstract

**Background:**

Although numerous biological processes have been implicated in Alzheimer's disease (AD) pathogenesis, plasma biomarkers have been largely limited to measures of amyloid‐b and *p*‐tau. We used a large‐scale plasma and brain tissue proteomic analyses to (i) identify early plasma biomarkers of AD and (ii) assess the mechanistic relevance of identified proteins.

**Method:**

We applied the SomaScan proteomic platform to measure the abundance of 4,877 plasma proteins among middle‐aged adults in the ARIC study. Dementia was assessed over the subsequent 25‐year period. Cox proportional hazards models adjusted for demographic characteristics and cardiovascular risk factors were used to relate each plasma protein to 25‐year dementia risk. Using brain tissue proteomic results from the ROSMAP cohort, we (i) examined the extent to which identified proteins were differentially expressed in AD, (ii) identified brain tissue protein quantitative trait loci (pQTL), and (iii) used two‐sample Mendelian randomization to assess the causal link between candidate plasma proteins and AD dementia.

**Result:**

In proteome‐wide analyses of 10,981 adults (baseline age: 60 (SD 6); 21% Black; 54% women), we identified 32 plasma proteins associated with subsequent dementia risk, most of which were involved in biological processes such as proteostasis, immunity, extracellular matrix organization, and synaptic function (CPLX1, CPLX2, CBLN4). Synaptic proteins CPLX1 and CPLX2 were upregulated in plasma among those at risk for dementia over a 25‐year follow‐up period, whereas CBLN was down‐regulated among individuals at risk for dementia over a 15‐year follow‐up period. While the majority of the 32 dementia‐associated proteins were expressed across multiple tissue, synaptic proteins were primarily expressed in the central nervous system (CNS), and each synaptic protein was down‐regulated in AD brains, compared to control brains. Brain tissue pQTLs were identified for CPLX1. Two‐sample Mendelian randomization supported the causal link between brain CPLX1 levels and AD dementia (Z=2.13; *p* = 0.03).

**Conclusion:**

These results suggest that synaptic proteins are released from the CNS into the blood well before symptom onset. Synaptic proteins measured in blood, such as CPLX1, may represent mechanistically relevant biomarkers of AD dementia risk.